# Persistent pulmonary hypertension of the newborn infant (PPHN) due to premature closure of the ductus arteriosus (DA)

**DOI:** 10.1515/crpm-2024-0001

**Published:** 2024-04-29

**Authors:** Ayevbekpen Grace Okoye, Adrita Khawash, Mahesh Nanjundappa, Matthew Jones, Anne Greenough

**Affiliations:** Neonatal Intensive Care Centre, Kings College Hospital NHS Foundation Trust, London, UK; Paediatric Cardiology Department Evelina Children’s Hospital, Guys and St Thomas Hospital, London, UK; Department of Women and Children’s Health, School of Life Course Sciences, Faculty of Life Sciences and Medicine, King’s College London, London, UK

**Keywords:** persistent pulmonary hypertension of the newborn, ductus arteriosus

## Abstract

**Objectives:**

To describe the course of an infant with persistent pulmonary hypertension of the newborn (PPHN) secondary to premature closure of the ductus arteriosus (DA), a very rare phenomenon which can lead to adverse clinical outcomes.

**Case presentation:**

A term infant was diagnosed with severe PPHN with echocardiographic features noted at 6 h after birth which included supra-systemic pulmonary pressures, severe isolated right ventricle (RV) hypertrophy, poor RV dysfunction and no ductal flow in the context of a structurally normal heart. There was maternal use of low-dose aspirin in pregnancy due to preeclampsia. There is a known association between use of prostaglandin synthase inhibitors such as aspirin with ductal closure leading to increased RV pressure. Treatment was commenced with positive pressure ventilation, inhaled nitric oxide (iNO) and milrinone. There was a limited response to iNO necessitating increasing the concentration of milrinone with a marked improvement in oxygenation. Following commencement of sildenafil, inhaled nitric oxide was gradually weaned and stopped in the third week and the infant extubated. The infant was discharged home on oral sildenafil at four weeks of age with no respiratory or feeding support. Echocardiographic features of raised right sided pressures persisted, but with reduced RV hypertrophy and septal flattening and improved RV function. Oral sildenafil was subsequently weaned and stopped at four months of age.

**Conclusions:**

A severe form of PPHN due to premature closure of the DA requires early discussion with the cardiologist. The use of milrinone and sildenafil can lead to a favourable outcome.

## Introduction

**Figure 1:**
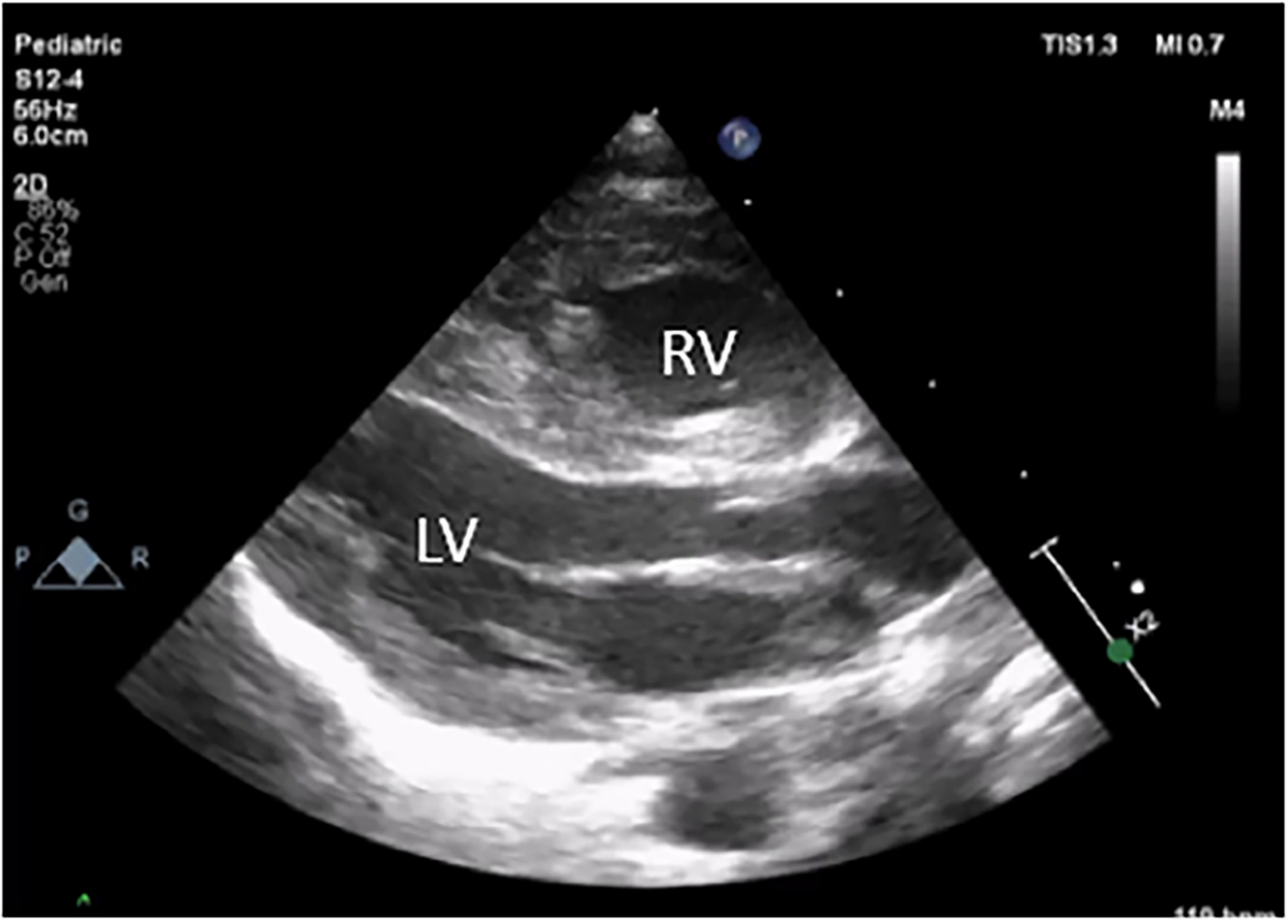
Echocardiogram parasternal long axis view demonstrating severe right ventricular hypertrophy (RVH). RV, right ventricle; LV, left ventricle.

**Figure 2:**
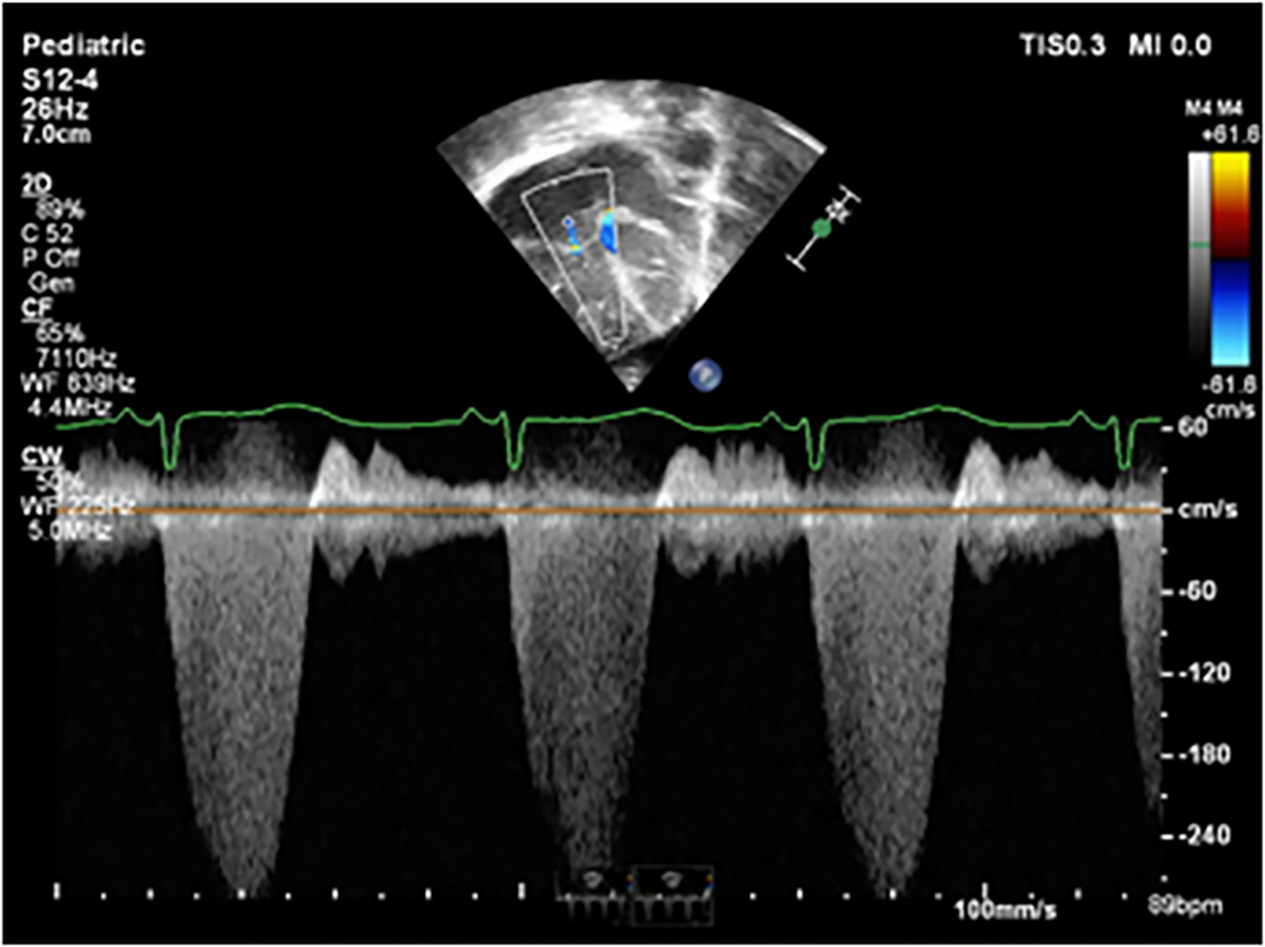
Echocardiogram continuous wave Doppler demonstrating severe tricuspid regurgitation 3.5 m/s.

Persistent pulmonary hypertension of the newborn due to premature closure of the ductus arteriosus is a rare phenomenon which can lead to significant cardiovascular changes and pose a diagnostic and treatment challenge. Clinical presentations and outcomes have marked variability and reversibility of effects with a spectrum of clinical sequelae [1]. There is a known association of maternal exposure to prostaglandin synthase inhibitors. Premature closure of the ductus arteriosus causes volume overload on the fetal pulmonary circulation which can lead to pulmonary hypertension [2]. We present an infant with severe persistent pulmonary hypertension of the newborn (PPHN) due to premature closure of ductus arteriosus (DA) who was successfully treated with milrinone and sildenafil (Figures 1 and 2).

## Case presentation

A term infant with a birth weight of 3.4 kg was delivered to a healthy 32 year old woman at 40+6 weeks of gestation via emergency caesarean section due to a pathological cardiotocography (CTG). There was maternal history of exposure to low dose aspirin (150 mg daily) in pregnancy due to a previous history of pre-eclampsia. There was reported mild polyhydramnios at 34 weeks of gestation but otherwise the antenatal ultrasound examinations showed no abnormality. There were no maternal risk factors for sepsis and no meconium-stained liquor. Shortly after birth, the infant developed a significant oxygen requirement needing intubation and ventilation. The ventilation mode was volume guarantee with the volume targeting set at 6 mL/kg, PEEP 5 cmH_2_O, peak pressure set at 30 cmH_2_O, inspiration time of 0.45 s and the fraction of inspired oxygen of 1.0. Due to significant hypoxia and hypoxaemia, the infant was given a neuromuscular blocking agent and sedation. Inhaled nitric oxide (iNO) of 20 ppm was commenced. Umbilical venous and arterial catheters were inserted. There was a pre and post oxygen saturation difference of more than 5 %. The first arterial blood gas showed a ph of 7.286, pO_2_ of 6 kPa and pCO_2_ of 4.38. Inotropic support with dopamine and noradrenaline was commenced to optimise the systemic blood pressure.

Echocardiogram (ECHO) at 6 h of life showed severe PPHN with a structurally normal heart, no ductal flow, right ventricle (RV) dysfunction and isolated severe right ventricular hypertrophy (RVH) with no evidence of right ventricular outflow tract obstruction. In view of unusual presentation of PPHN and the echocardiographic (ECHO) findings, early discussion with cardiologist was initiated. The recommendation was to treat as severe PPHN due to premature closure of the ductus arteriosus (DA) and to continue noradrenaline and add milrinone infusion at 0.5 mg/kg/min because of the RV dysfunction. The chest radiograph on day one showed cardiomegaly with right upper lobe collapse. The arterial blood gas after commencing milrinone showed an improvement in the partial pressure of oxygen with ph 7.36, pO_2_ 17.7, pCO_2_ 7.23, base deficit −4.9. Due to reduced oxygenation and persistent evidence of severe PPHN on the echo on day five, the concentration of milrinone was increased from 0.5 to 0.75 μg/kg/min following advice from the cardiologist. The infant continued to receive mechanical ventilation with inhaled nitric oxide (iNO) and milrinone and noradrenaline for the first 16 days after birth. Improvement in oxygenation was refractory to inhaled nitric oxide, hence iNO was weaned slowly and based on the PaO_2_ and the inspired oxygen requirement. Treatment with iNO was complicated by methylhaemoglobinaemia, which required use of methylene blue infusion on day 10. The maximum methaemoglobin level reached was 3 %. On day 11, the cardiologist recommended commencement of a low dose of sildenafil (0.25 mg/kg/dose four times daily) and to monitor the blood pressure, then gradually to increase the dose over next 72 h to 1 mg/kg/dose four times daily. In view of poor RV function, milrinone infusion was slowly weaned and stopped on day 18. The addition of sildenafil resulted in a marked and sustained rise in paO_2_ with maximum levels being a paO_2_ of 22. On day 14, the echocardiographic features were much improved with flow across the interatrial septum now bidirectional and improved RV function with better filling. On day 17, the infant was extubated onto high flow oxygen and subsequently, after 24 h, was self-ventilating in room air.

Further investigations following the cardiologist’s advice were undertaken to rule out other potential causes of pulmonary hypertension of this severity. Ultrasound of the liver and brain were done to determine if there were arteriovenous malformations and genetic studies were sent for pulmonary hypertension. There was a further plan to consider computerised tomography of the pulmonary arteries (CTPAs) if there was no improvement in clinical status. Subsequent echocardiograms showed persistent features of raised pulmonary pressures, so sildenafil was continued up to a maximum dose of 1 mg/kg four times daily. On day 28, evidence of elevated right sided pressures persisted but with reduced RV hypertrophy and improved RV function. The infant was discharged home with oral sildenafil. Sildenafil was subsequently weaned and stopped at four months with normal echo findings. Magnetic resonance imaging (MRI) of the brain was normal.

## Discussion

We report an infant successfully treated for PPHN due to premature closure of the DA which is rare. Indeed, there is no known documented case of a term infant treated for signs of premature closure of DA with such a favourable outcome. The clinical findings demonstrated in this case were consistent with a diagnosis of persistent pulmonary hypertension of the newborn. Echocardiographic features demonstrated isolated RV hypertrophy and absence of ductal flow within the first 6 h, evidence of premature closure of the duct. In addition to all those features presenting as postnatal hypoxaemia, there was absence of significant lung disease and any structural heart defect. We were able to successfully treat this infant with iNO, milrinone and sildenafil.

PPHN secondary to premature closure of the DA is a rare with marked variability in presentation, reversibility of effects and outcomes [1]. Information on the incidence and long term outcome are limited with some cases of pulmonary hypertension and fetal death being reported [3]. There is significant RV hypertrophy in all cases [4]. There is a well-documented link between closure of the DA and the echocardiographic features seen in this case. The DA controls the RV output *in utero*. Closure of the DA leads to fetal pulmonary overcirculation which leads to increased muscularisation of pulmonary vasculature. The result is the RV is contracting against high pressure overtime leading to hypertrophy, RV overload and dysfunction. DA constriction or closure has been associated with maternal use of non-steroidal anti-inflammatory drugs (NSAID) in pregnancy [5, 6], although a recent study demonstrates no conclusive association [7].

This is a rare case with no documented standardised management. Clinical presentation is variable with reported cases of fetal demise, hydrops and management with ECMO due to refractory hypoxaemia [1, 8]. In this case, early discussion with the cardiologist and recommendations on the use of milrinone, iNO and sildenafil proved beneficial with a successful outcome.

## Take home message of the lessons learnt

Persistent pulmonary hypertension of the newborn due to premature closure is very rare with variable presentation and mostly poor outcomes documented in the literature.

Early discussion with a cardiologist with regards to unusual echocardiographic findings is vital to reach a diagnosis and appropriate management. The use of milrinone and sildenafil may make a difference to the outcome.
